# Joint modeling of time to diabetic retinopathy and change in fasting blood sugar among type 2 diabetic patients, Northwest Ethiopia

**DOI:** 10.1038/s41598-022-06240-5

**Published:** 2022-02-09

**Authors:** Sewnet Adem Kebede, Zemenu Tadesse Tessema, Shitaye Alemu Balcha, Tadesse Awoke Ayele

**Affiliations:** 1 Department of Epidemiology and Biostatistics, Institute of Public Health, College of Medicine and Health Sciences, University of Gondar, Gondar, Ethiopia; 2 Department of Internal Medicine, School of Medicine, College of Medicine and Health Sciences, University of Gondar, Gondar, Ethiopia

**Keywords:** Endocrinology, Medical research, Risk factors

## Abstract

This study aimed to assess changes in fasting blood sugar (FBS) levels, time to diabetic retinopathy (DR) and its predictors among type 2 diabetes patients in Ethiopia. An institution-based retrospective follow-up study was conducted at the University of Gondar Comprehensive Specialized Hospital. The linear mixed effect model and Cox proportional hazard models were fitted separately, and later, the two models were fitted jointly using R software. Variables with a *p* value < 0.05 were considered significant predictors in the adjusted analysis. The incidence rate of DR was 2 per 100-person year of observation with a median follow-up time of 90.8 months (IQR 63.4). The current value and rate of change in FBS level were significant predictors of time to DR (AHR = 1.35; 95% CI 1.12–1.63) and (AHR = 1.70; 95% CI 1.21–2.39), respectively. Hypertension (AHR = 2.49; 95% CI 1.32–4.66), taking > 1 antidiabetic oral agent (AHR = 4.90; 95% CI 1.07–20.0) and more than 10 years duration (AHR = 0.17, 95% CI 0.06–0.46) were predictors of time to DR. This study revealed that the current value of FBS and the rate of FBS change were significantly associated with the time to DR.

## Introduction

Diabetes mellitus (DM) is a metabolic disorder of multiple etiologies characterized by chronic hyperglycemia with disturbances of carbohydrate, fat and protein metabolism resulting from defects in insulin secretion, insulin action or both^1,2^.

Type 2 diabetes mellitus is the most common form and covers 90% of people with diabetes around the world^2–4^. Type 2 DM patients are more susceptible to various forms of complications, such as cardiovascular disease, neuropathy, nephropathy and eye disease, leading to retinopathy and blindness^3,5,6^.

Diabetes mellitus has a long-term effect on retinal small blood vessels, which cause progressive development of the specific complications of retinopathy, resulting in vision loss ^1,7^. Diabetic retinopathy (DR) is the leading cause of vision loss in working-age adults (20 to 65 years), and approximately one in three people living with diabetes have some degree of DR, and one in ten will develop a vision-threatening form of the disease^3^. Globally, the number of people with DR will grow from 126.6 million in 2010 to 191.0 million by 2030, and it is estimated that the number with vision-threatening DR will increase from 37.3 million to 56.3 million if prompt action is not taken^8^.

The magnitude of DR in Africa ranges from 30.2 to 31.6% and 7.0 to 62.4% in patients with diabetes in population-based studies and clinic-based surveys, respectively^9^. A population-based study in Kenya identified a prevalence of DR 35.9 in 277 people with diabetes^10^. In Ethiopia, the magnitude of DR ranged from 5.0% in northwest Ethiopia to 43.4% for T2DM patients in southwest Ethiopia. Many studies performed in Ethiopia have shown that the presence and severity of complications related to DR are steadily increasing^11^.

Risk factors for DR among diabetes patients include sex, uncontrolled DM, longer duration of DM, presence of nephropathy and presence of hypertension, which have been documented in different studies^12–15^.

Until an advanced stage, DR is asymptomatic and is largely preventable through regular retinal screening and prompt treatment before visual deterioration. However, screening for DR is not performed as expected for diabetes patients in resource-limited areas because it is not easily accessible and affordable.

Therefore, monitoring a longitudinal marker is essential to be aware of metabolic abnormalities and poorly controlled blood sugar, which increase the likelihood of retinopathy occurring and cause retinopathy to progress more quickly and provide meaningful prognostic information that can help to differentiate patients with regard complications and lead to changes in other interventions.

Previous studies conducted in Ethiopia used separate analyses, which ignored the dependency and association between longitudinal FBS and time to DR. A joint modeling approach is preferred over a separate survival model, as it provides more efficient estimates of the treatment effects on the time to DR and longitudinal marker of FBS measured with error. Hence, this study uses a joint modeling approach to account for the dependence and association of time to DR and FBS change.

## Methods

### Study design and area

An institutional-based retrospective follow-up study was conducted from January 2001 to February 2016 among type 2 diabetes patients at the University of Gondar Comprehensive Specialized Hospital (UGCSH). UGCSH serves more than five million people of the North Gondar zone and people of the neighboring zones. Approximately 24,862 people have chronic follow-up per year, and among these, 8,900 are DM patients.

### Study population

All newly diagnosed type 2 diabetes patients at UGCSH from January 2001 to February 2016 were included in the study. However, those whose date of initiation was not recorded, those who had only one FBS measurement and newly diagnosed patients who had DR at the start of follow-up were excluded.

### Sample size determination and sampling procedure

The sample size was determined for the two processes: longitudinal and survival.

### Sample sized for longitudinal outcome

The sample size was determined by considering the following statistical assumptions: two-sided significance level (α = 5%), power 80%, number of repeated measurements (m = 7), within subject correlation (*ρ* = 0.5) and effect size ($\delta =0.0384$) using n=$\frac{{2\left({z}_{\frac{\alpha }{2}}+{z}_{\beta }\right)}^{2}(1+(m-1)\rho }{{m\delta }^{2}}$^16^. The effect size ($\delta$) and number of repeated measurements (m) for calculating the sample size in this study were taken from a study in Debre Berhan referral hospital, Ethiopia^17^, and the calculated sample size was 466.

### Sample sized for survival outcome

To estimate the survival probability of patients, we considered sex as exposure based on research conducted in Tikur Anbessa Hospital in Addis Ababa^18^. Using STATA 14.1 by using power analysis for log rank test and considering Z value at 95% confidence, Power 80% and 10% incompleteness, the sample size was 435.

Finally, largest sample size (466) was selected. With 5% contingency, the final sample size required for this study was 489. After the patients fulfilled the selection criteria, study subjects were selected by using a systematic random sampling technique.

### Variables

The response variable for the survival sub model was time from the date of diagnosis until the occurrence of either DR or censoring measured in months, while the response variable for longitudinal sub model was blood glucose level in terms of FBS using mill gram per deci liter (mg/dL) from treatment start (baseline) and repeatedly measured every three months, were time varying endogenous covariate. Sociodemographic variables: baseline age (year), current age (year) and gender. Clinical variables: blood glucose, lipid level profile, body mass index (BMI), albuminuria level, diabetic duration, treatment type and creatinine clearance Comorbidities: hypertension, neuropathy, nephropathy and anemia were independent variables.

### Variable definition

For this study, diabetic retinopathy, time to diabetic retinopathy, censoring, poor glycemic control and hypertension were defined as follows: *diabetic retinopathy* was established if the subject had a minimum of one microaneurysm in any field, showed hemorrhages (dot & blot, or flame shaped), or maculopathy (with or without clinically significant edema)^14^. *Time to diabetic retinopathy* is the time between the time of diagnosis of T2DM and the development of diabetic retinopathy. *Censored* data included loss to follow-up, death and being event free at the end of the study. *Hypertension was* diagnosed when SBP was greater than or equal to 140 mmHg and DBP was greater than or equal to 90. *Poor glycemic control* was established if fasting blood sugar was greater than or equal to 140 mg/dL.

### Data collection and data quality control

Sociodemographic characteristics and baseline and follow-up clinical and laboratory data collected from patient cards. Diabetic retinopathy was identified based on a history of diabetes mellitus and fundoscopy findings. Diabetic retinopathy grading in this study was described based on DR screening and treatment guidelines^19^. A week before the actual data collection, a preliminary review was performed in a similar area.

### Statistical analysis

Descriptive measures such as the means, medians, IQRs, percentages, frequencies and standard deviations were used to describe the study population. The survival experience of the patients was assessed using Kaplan–Meier survivor function. The log rank test was used to compare the survival experiences among the different groups of subjects. Cox PH and three parametric models (Weibull, exponential and log logistic) were fitted to identify the risk factors. The best model was selected by using Akaike information criteria (AIC). Accordingly, the Cox PH model was the preferred model to model time to DR among type 2 DM patients in the study area. Schoenfeld residuals tests and graphical methods were used to check the Cox proportional hazard (PH) assumption before fitting the survival sub model. The goodness of fit of the model was assessed by using the Cox-Snell residual technique.

To account for the effect of an endogenous time-varying covariate (FBS) on the time to DR, the true unobserved value of fasting blood sugar in the survival model was used. The trajectory FBS over time was approximately normally distributed. Exploratory analysis was used to visualize the patterns of individual profile plots and average evolution changes graphically. A linear mixed effect model with random intercept only and both random intercept and slope was fitted. A linear random effect model with both intercept and slope was the best model that appropriately predicted the mean change in FBS measurements over time. To estimate the effects of longitudinal fasting blood sugar change on the risks of DR, the complete true history of fasting blood sugar for each subject was determined using a linear mixed effect model constructed by considering the effects of baseline covariates on fasting blood sugar evolution. The association parameter (alpha value) from the fitted joint model was used to assess the association between longitudinal marker (fasting blood sugar) and time to DR.

## Model specification

### Linear mixed modeling

Linear mixed models are a type of regression model that take into account both variation that *is* explained by the independent variables of interest and variation that is *not* explained by the independent variables of interest. This is due to the measurement taken from the same subject at different time points or the measurements taken from the same clusters are likely to be correlated. In this study, before joint modeling to have an appropriate longitudinal sub model for longitudinally measured fasting blood sugar, LMM was employed to identify the covariates that had significant effects on the mean change in FBS measurements over time. Therefore, longitudinal data modeling began with exploratory data analysis to determine the mean change in FBS measurement over time. To measure the effect of the longitudinal covariate on the risk for an event, we need to estimate the true unobserved value of the longitudinal covariate ${m}_{i}\left(t\right)$ and successfully reconstruct the complete longitudinal history ${M}_{i}\left(t\right)$ for each subject. To achieve this, we postulate a suitable mixed-effects model to describe the subject-specific time evolutions with this notation

$$ \begin{aligned} & y_{i} \left( t \right) = m_{i} \left( t \right) +\upvarepsilon _{i} \left( t \right) \\ & m_{i} \left( t \right) = x_{i} \left( t \right)\beta_{1} + z_{i} \left( t \right)b_{i} \\ & b_{i} \sim N\left( {0,\Sigma_{i} } \right),\upvarepsilon { }_{i} \sim N\left( {0, \sigma^{2}_{i} } \right) \\ \end{aligned} $$

where we explicitly note that the design vectors ${X}_{i}\left(t\right)$ for the fixed effects β and ${Z}_{i}\left(t\right)$ for the random effects ${b}_{i}$, as well as the error terms $\upvarepsilon _{i} \left( t \right)$, are time dependent. We assume that error terms are mutually independent, independent of the random effects, and normally distributed with mean zero and variance σ^2^.

### Survival sub modeling

The hazard function of the survival model is used to explain the probability that the event has occurred by duration t. This study considered semiparametric survival models to explain how the risk or hazard of DR occurring at a given time is affected by covariates in the study area. The Cox proportional hazard model expresses the hazard of an event at time *t* as:

$${\lambda }_{i}\left(t\right)={\lambda }_{o}\left(t\right)exp\left({W}^{T}\upgamma \right)$$

where W is the matrix of baseline covariates, $\upgamma$ is the vector of parameters and the term ${\lambda }_{o}$ is the baseline hazard where the effects of covariates are zero.

### Joint modeling structure

A joint model of longitudinal and time-to-event data can effectively assess the impact that a longitudinal covariate, measured with error, has on the time to an event of interest. In this study, we assessed the predictive ability of a longitudinal marker, FBS, on time to DR. To introduce this methodology, we use the following notation to harmonize diverse approaches.

In particular, we denote by ${{T}_{i}}^{*}$ the true event time for the $i$ ^th^ subject, ${T}_{i}$ the observed event time, defined as the minimum of the potential censoring time ${C}_{i}$ and ${{T}_{i}}^{*}$, and by ${\delta }_{i}$ = $I\left({{T}_{i}}^{*}\le {C}_{i}\right)$ the event indicator. For the endogenous time-dependent covariate (FBS), we let ${y}_{i}\left(t\right)$ denote its observed value at time point $t$ for the $i$ ^th^ subject. We should note that we do not actually observe ${y}_{i}\left(t\right)$ for any time $t$ but rather only at the very specific occasions ${t}_{ij}$ at which measurements were taken. Thus, the observed longitudinal data consist of the measurements

$${y}_{ij}=\left\{{y}_{i}\left({t}_{ij}\right),j=1,\dots ,{n}_{i}\right\}$$

The term ${m}_{i}\left(t\right)$ denotes the true and unobserved value of the longitudinal outcome at time t. Note that ${m}_{i}\left(t\right)$ is different from ${y}_{i}(t)$, with the latter being contaminated with the measurement error value of the longitudinal outcome at time $t$. To quantify the strength of the association between ${m}_{i}\left(t\right)$ and the risk for an event, a straightforward approach is to postulate a relative risk model of the form**:**

1 $$ \left\{ \begin{gathered} h_{i} \left( {t\backslash M_{i} \left( t \right),w_{i} } \right) = lim_{dt \to 0} {\text{ Pr}}\left( {{ }\frac{{{\text{t}} \le T_{i}^{*} \left\langle {{\text{t }} + {\text{dt}}\backslash T_{i}^{*} } \right\rangle {\text{t}},M_{i} \left( t \right),{\text{ wi}}}}{dt}} \right) \hfill \\ \quad h_{i} \left( {t\backslash M_{1i} \left( t \right),w_{i} } \right) = { }h_{0} \left( {\text{t}} \right){\text{ exp}}\left\{ {\gamma^{T} W_{i} + \alpha_{1} m_{1i} \left( t \right)} \right\},t > 0 \hfill \\ \end{gathered} \right. $$

where ${M}_{i}\left(t\right)$ = {${m}_{i}\left(s\right)$,0 ≤ s < t} denotes the history of the true unobserved longitudinal process up to time point t, ${h}_{0}\left(.\right)$ denotes the baseline risk function and ${W}_{i}$ is a vector of baseline covariates with a corresponding vector of regression coefficients γ. Similarly, parameter ${\alpha }_{1}$ quantifies the effect of the underlying longitudinal outcome on the risk for an event.

2 $${h}_{i}\left(t\right)={h}_{o}\left(t\right) exp\left\{{\gamma }^{T}{w}_{i}+{\alpha }_{1}{m}_{i}\left(t\right)+{\alpha }_{2}{m}_{i}^{^{\prime}}\left(t\right)\right\}$$

where ,$${m}_{i}^{\mathrm{^{\prime}}}\left(t\right)=\frac{d}{{d}_{t}}{m}_{i}\left(t\right)=\frac{d}{{d}_{t}}\left\{{x}_{i}^{T}\left(t\right)\beta +{z}_{i}^{T}\left(t\right){b}_{i}\right\}$$

${\alpha }_{2}$ measures how strongly associated the value of the slope of the true longitudinal trajectory at time t is with the risk for an event at the same time point, provided that ${m}_{i}\left(t\right)$ remains constant. The relative risk model (1) postulates that the risk for an event at time $t$ depends only on the current value of the time-dependent marker ${m}_{i}\left(t\right)$. The relative risk model (2) postulates that the risk depends on both the current true value of the trajectory and the slope of the true trajectory at time t^20^.

### Ethical consideration

Ethical clearance was obtained from the Institutional Review committee of the University of Gondar with reference number / IPH/180/06/2011. Then, permission letters from officials of University of Gondar Comprehensive Specialized Hospital, Department of Internal Medicine were processed before data collection in order to access medical records of patients. Informed consent is not required as the whole data had been retrieved from the medical records of patient. An informed consent was waived by Institutional Review committee of the University of Gondar. All methods were performed in accordance with the relevant guidelines and regulation of declaration of Helsinki Data were fully anonymized and no personal identifiers, such as name and private information were not collected. Confidentiality during all phases of research activities was kept and data were held on secured password protected system.

### Ethics approval and consent to participate

Ethical clearance was obtained from the Institutional Review committee of the UOG with reference number / IPH/180/06/2011. Informed consent was not required, as all data were retrieved from the medical records of the patients. Data anonyms and hold on a secure password protected system. Confidentiality during all phases of research activities was maintained.

### Consent for publication

Not applicable.

## Results

### Descriptive statistics

Among the total type 2 DM patients during the time period, 80 (17.17%) developed DR. The mean age of participants at baseline was 53.17 (SD = 10.11) years, and of the total study subjects, 69.74% were within the age category < 60 years. The mean duration of diabetes was 9.2 years (SD =  ± 3.8). Among 249 T2DM patients whose baseline FBS was above 200 mg/dl, 55% developed DR. Out of a total of 78 patients who developed neuropathy, 22.5% developed DR (Table 1).

**Table 1:** Demographic and clinical characteristics of the study participants in UGCSH, 2001–2016.

Variable	Censored (%)	Diabetic retinopathy (%)	Total
**Sex**			
Male	153 (39.64)	34 (42.50)	187 (40.13)
Female	233 (60.36)	46 (57.50)	279 (59.87)
**Baseline age**	Mean = 53.17 ± 10.11		
< 60	266 (68.91)	59 (73.75)	325 (69.74)
≥ 60	120 (31.09)	21 (26.25)	141 (30.26)
**Residence**			
Gondar	309 (80.05)	69 (86.25)	378 (81.12)
Out of Gondar	77 (19.95)	11 (13.75)	88 (18.88)
**Baseline FBS**	Mean = 224.3 ± 81.76		
70–130	33 (8.55)	2 (2.50)	35
131–200	148 (38.34)	34 (42.50)	182
> 200	205 (53.11)	44 (55)	249
**HTN**			
Yes	79 (20.49)	29 (36.25)	108
No	307 (79.53)	51 (63.75)	358
**Medication**			
No medication	25 (6.48)	4 (6.25)	29
1 oral agent	247 (63.9)	51 (62.5)	298
> 1 oral agent	68 (17.6)	12 (15)	80
Insulin	46 (11.9)	13 (16.3)	59
**Neuropathy**			
Yes	60 (15.5)	18 (22.5)	78
No	326 (84.5)	62 (77.5)	388
**Nephropathy**			
Yes	55 (14.25)	52 (15)	67
No	331 (85.75)	28 (85)	399
**Anemia**			
Yes	31 (8.03)	7 (8.75)	38
No	355 (91.97)	73 (91.25)	428
**Duration**	Mean = 9.2 ± 7.7		
< 6 years	82 (21.2)	18 (22.5)	100
6–10 years	174 (45.08)	31 (38.75)	205
> 10 years	130 (33.68)	31 (38.75)	161
**Baseline SBP**			
≤ 140	308 (79.79)	69 (86.25)	377
> 140	78 (20.21)	11 (13.75)	89
**Baseline DBP**			
≤ 90	344 (89.12)	72 (90)	416
> 90	42 (10.88)	8 (10)	50

### Exploring fasting blood sugar change

**Figure 1:**
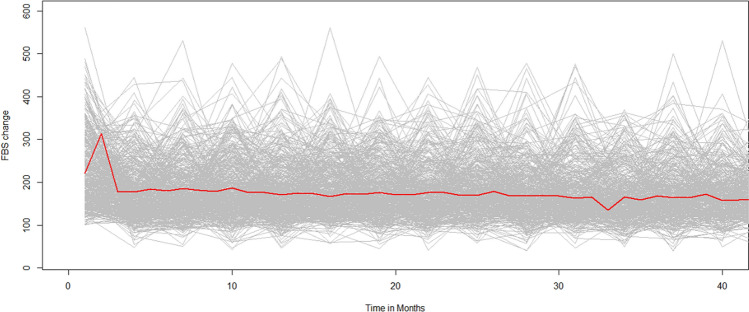
The individual profile plots suggested that there were within- and between-variation changes in FBS measurements over time. The individual trajectory of FBS for patients shows that patients had considerably different FBS at baseline and over time. This suggests that a model with both a random intercept and random slope is required. The red line (loess smoothing technique), which shows the mean structure of FBS measurement over time, suggested a linear change in the mean FBS measurement over time.

To understand the association between the FBS measurement and time, individual profile plots were employed. The loess smoothing technique over individual profile plots was used to explore the mean change in FBS measurement over time (Fig. 1).

As indicated on the plots, the individual profile plots suggested that there were within- and between-variation changes in FBS measurements over time. The individual trajectory of FBS for patients shows that patients had considerably different FBS at baseline and over time. This suggests that a model with both random intercept and slope is a good start. However, the red line, which shows the mean structure of FBS measurement over time with the loess smoothing technique, suggested a linear change in the mean FBS measurement over time.

### Sensitivity analysis

When FBS was not observed during a 3-month period, values were considered missing. A problem in the handling of missing data in longitudinal outcomes is the fact that the observed data alone cannot distinguish between an MAR and an MNAR dropout mechanism. To handle this, we perform sensitivity analysis and then analyze the sensitivity of the reported results by observing the parameter estimate and the standard error estimate. The parameter estimate between complete case analysis and multiple imputation is not that much different. Therefore, by considering this joint model with the current parametrization with a complete case analysis approach, the model was chosen (Table 2).

**Table 2:** Comparison of the joint model with the current value and slope parameterization under multiple imputation and complete case analysis missing handling method for T2DM patients.

Method	Parametrization	AIC
Multiple imputation	Current	68,944
	Slope	68,830
Complete case analysis	Current	27,625
	Slope	27,719

AIC agrees that the joint model with the current parametrization with a complete case analysis missing handling approach has a better predictive ability.

### Time to retinopathy

**Figure 2:**
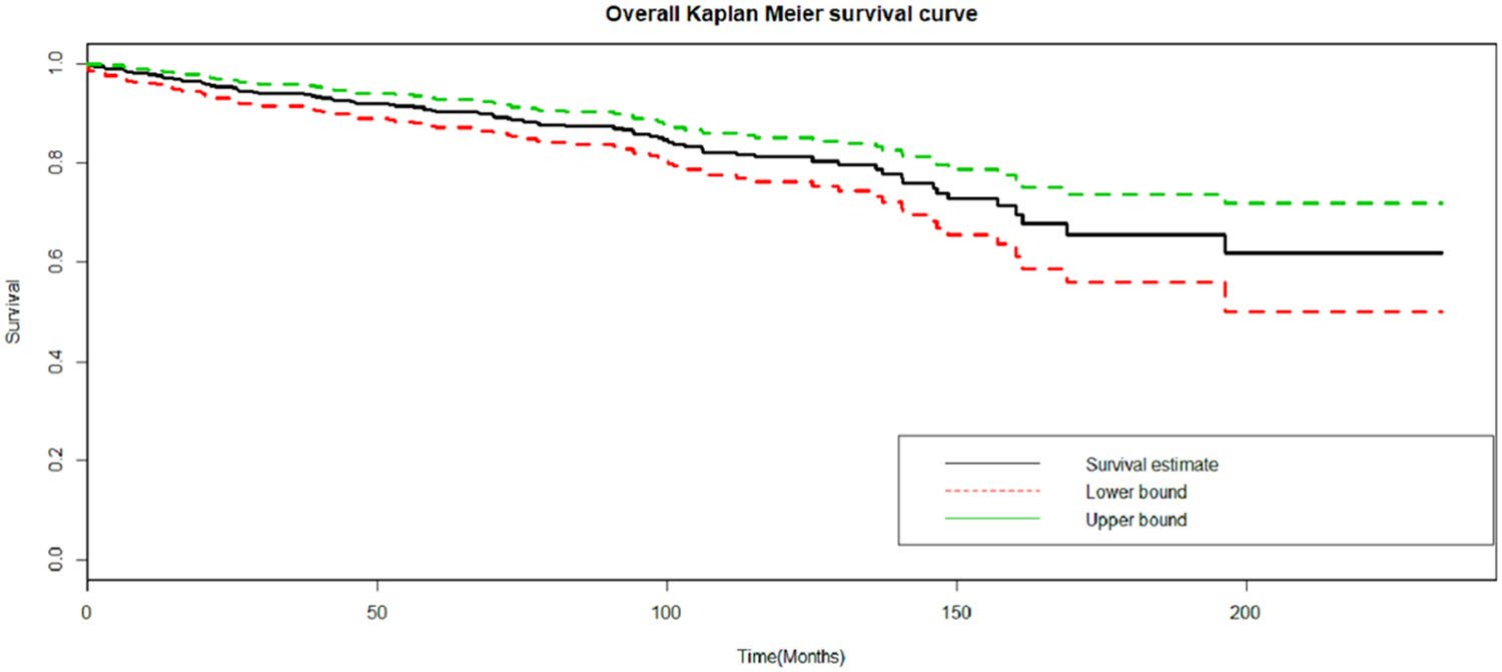
The plot of the overall estimate of Kaplan–Meier survivors for time to DR among T2DM patients under anti-diabetes treatment at UGCSH, 2001–2016. The black line indicates the survival estimate, and the red and green line show the lower and upper boundaries of the estimate. The median survival time or the survival time at which the cumulative survival function was equal to 0.5 could not be determined.

The cumulative survival probability of the patients at the end of 12, 36 and 72 months were 97%, 94%, and 89%, respectively. The cohort contributed a total of 3607.67 person-years. Approximately half (51%) of DR develops in the first five years. The median follow-up time was 90.8 [IQR = 63.4] months, and a total of 80 (17.17%) patients developed DR. The incidence density was 2 cases per 100 patients per year (95% CI 1.78 2.76). The median survival time or the survival time at which the cumulative survival function was equal to 0.5 could not be determined (Fig. 2).

### Joint model

After the most suitable separate model was decided for the data, the proposed joint model was applied with the aim of investigating the effects of FBS measurements on time to DR.

The joint model was fitted by both current value- and time-dependent slope parameterization with the piecewise-PH-aGH method to specify the type of survival sub model and the algorithm numerical integration method, among the available options. This result validates the hypothesis that for type 2 DM patients, the current value of FBS and the slope of the FBS trajectory are highly associated with the hazard of the event (diabetic retinopathy).

In this study, a survival analysis-longitudinal analysis joint modeling technique was used to identify the risk factors for the time to the occurrence of DR and changes in longitudinal FBS. The type of medication, duration, weight, current age, and time were found to be significant predictors of changes in FBS, whereas the presence of hypertension, medication and duration were significant predictors of time to DR (Table 3).

**Table 3:** The fitted joint model result under current parameterizations for type 2 diabetes patients in UGCSH, 2001–2016.

Longitudinal sub model			Survival sub model		
Fixed effects	Coeff (β)	95% CI	Covariates	AHR	95% CI
			Sex		
Intercept	13.50	[11.46 15.55]***	Male	1	
FBS time 1	− 0.288	[− 0.93 0.35]	Female	0.83	[0.43 1.59]
FBS time 2	− 4.013	[− 4.70 − 3.32]***	HTN		
FBS time 3	− 1.444	[− 1.92 − 0.96]***	No	1	
Baseline SBP			Yes	2.49	[1.32 4.66]**
≤ 140	0		Medication		
> 140	0.045	[− 0.51 0.60]	No medication	1	
Current Age	− 0.255	[− 0.49 − 0.02]***	1 oral agent	2.55	[0.43 7.38]
Medication			> 1 oral agent	4.90	[1.07 20.0]*
Dietary modification	0		Insulin	4.28	[0.78 21.2]
1 oral agent	0.547	[− 0.32 1.41]	Neuropathy		
> 1 oral agent	0.902	[0.21 1.59]**	No	1	
Insulin	0.701	[− 0.18 1.58]	Yes	1.76	[0.81 3.81]
Sex			Nephropathy		
Male	0		No	1	
Female	0.130	[− 0.35 0.61]	Yes	1.47	[0.63 3.42]
Duration	0.096	[0.03 0.16]***	Duration		
Weight	0.034	[− 0.05 − 0.02]***	≤ 5.9 years	1	
**Variance component**			6–10 years	0.31	[0.11 0.84]*
Std. Dev. (intercept)	1.66		≥ 10 years	0.17	[0.06 0.46]***
Std. Dev. (Time)1	3.86		**Association parameters**		
Std. Dev. (Time)2	2.41		Parametrization		
Std. Dev. (Time)3	0.86		Current	1.35	[1.12 1.63]***
Std. Dev. (Residual)	3.17		Slope	1.70	[1.21 2.39]***

The hazard of DR for hypertensive patients is 2.49[1.32 4.66] times higher than those patients who were not hypertensive, keeping other variables constant. Unexpectedly, the hazard of developing DR among patients with a duration of 6–10 years is 69% less likely than patients with a duration of less than 6 years, keeping other variables constant. The hazard of developing DR among patients with a duration > 10 years is 83% less likely than that among patients with a duration less than 6 years, keeping other variables constant.

The hazard of developing DR for patients who take more than one oral antidiabetic agent together to manage their diabetes is 4.90[1.07 20.0] times higher than those patients who were not taking any medication while keeping other variables constant.

A unit increase in the current value of FBS increases the hazard of developing DR by 1.35 [95% CI 1.12 1.63] times, whereas a unit increase in the rate of FBS trajectory increased the hazard of DR by 1.70 [95% CI 1.21; 2.39] times, provided that the true an observed value remained constant (Table 3).

The joint model could be fitted like

$$\begin{aligned}{H}_{i}\left[t|{M}_{i}\left(t\right),{W}_{i}\right]&=0.91*hypertension-0.18*femalesex-0.20*baselineage\\&\quad -1.16*{duration}_{6-10}-1.75*duration\\&>10\,year+1.93*1\,oral\,agent+1.59*\\&>1\,oral\,agent+1.45*insulin-0.16*SBP0-0.002*{BMI}_{18.5-24.9}\\&\quad+0.37*{BMI}_{25-29.9}+0.14*{BMI}_{\ge 30}+0.30*{M}_{i}\left(t\right)\end{aligned}$$

where ${M}_{i}\left(t\right)$ is the true unobserved longitudinal process up to time t. ${W}_{i}$ is the vector of fixed effect covariates.

## Discussion

Diabetic retinopathy is a long-term complication of diabetes mellitus that could be attributed to systemic or local ocular factors^21^. The disease is asymptomatic until it leads to visual deterioration. Monitoring the longitudinal marker may be essential to be aware of the factors which increase the likelihood of retinopathy and to act accordingly. Joint modeling approach was applied in order to determine how strong is the association between FBS and the risk of DR.

Nearly half of DR were developed with in the first five years. This is consistent with the study done in Arbaminch^13^ which shows that 47% of DR developed with in the first five years. This could be because most type 2 diabetes patients only come to the health center when they have a complication, such as a vision impairment, because the disease is asymptomatic until it is advanced. However, this study finding is higher than the study done in Pakistan^22^ which showed that only 22% of DR developed with in the first five years.

The results of this study show that the proportion of DR was 17.17% with 20 cases per 1000 year. The study showed a higher incidence of DR than studies done in Ethiopia and China^18,23^. This inconsistency might be due to the difference in health care service and complication monitoring. However, the incidence rate in this study was lower than that of a study done in Ethiopia^13^ and Pakistan^22^. This discrepancy could be explained by differences in study period, diagnostic methods used in the studies, and the denominator population.

The current study showed that duration of diabetes was negatively associated with the hazard of DR. This finding is consistent with the study done in Arbaminch^13^ but it is inconsistent with the study done in Malaysia^24^, Ethiopia^25^, Pakistan^14^ and China^26^. This could be because our study participants were type 2 diabetes patients, who are more likely to come late to a health institution and seek medical attention because T2DM is a more gradual and less severe condition than type 1 diabetes. Another possible explanation for this finding, type 2 DM patients may gradually develop a better metabolic control with low levels of FBS.

In this study, we found that hypertension is a risk factors for DR. This finding is consistent with the studies from Ethiopia^13,27^ and also by UK Prospective Diabetes Study (UKPDS 38)^28^. Increased blood flow could damage the retinal capillary endothelial cells in eyes of people with diabetes^29^. The pathophysiological mechanism involves the function and interaction of the endothelial and vascular smooth muscle cells damage and altered growth of retinal capillary endothelial cells induce proliferative lesions of the retina^30^.

The current study showed that taking more than one oral anti diabetic agents together to manage their diabetes increased the risk of DR. This might be due to combination of oral agent is generally advocated when no medication or only one oral medication users had poorly control glycemia at some points and when patients develop resistance to medication which is expressed by poor glycemic control. Prolonged poor glycemia control causes injuries to the retinal vasculature. But other studies^13,18,24^ shows that medication has no association with risk of DR. So further investigation is needed.

In this study, we found strong evidence that both current value of FBS and rates of FBS change were associated with DR. Several earlier studies on type 2 DM patients showed that FBS level is significantly associated with DR^13,15,23,31^. But all these studies could not able to identify the effects of longitudinal FBS trajectory on the risk DR. By using joint modelling approach, we estimate the effects of longitudinal trajectory rate on the risk of DR.

One of the strengths of this study was the accuracy of DR since grading is not only a clinical ophthalmologic examination but also fundus photographs.

The main limitation of this study was that data on some potentially important predictors were not available, which may underestimate the effects and individual variations in the development of DR (some sociodemographic factors and behavioral factors). Another limitation was that in our study area, HBA1C was not performed for follow-up, which is the recommended mode of testing blood sugar control, leading to the use of FBS alone.

## Conclusion

This study revealed that the type of medication, duration, weight, current age, and time were significant predictors of changes in FBS, whereas the presence of hypertension, type of medication and duration were significant predictors of time to DR. Unobserved true current value of FBS and rate of FBS change were significantly associated with time to DR. Based on the study findings from these data, we recommend that it is better to screen for DR at the time of diagnosis of DM since most diabetes patients develop DR at the early period of the follow-up and to give special attention to those who took combined medication in the screening program. The health care provider strengthens the routine monitoring of FBS as a longitudinal marker that provides meaningful prognostic information that can help to differentiate patients with regard to DR and lead the way to change other interventions and close monitoring of patients diagnosed with hypertension.

## Data Availability

All necessary information was included in the manuscript.

## Abbreviations

AHR: Adjusted Hazard Ratio
AIC: Akaike Information Criteria
BMI: Body Mass Index
DBP: Diastolic Blood Pressure
DM: Diabetes Mellitus
DR: Diabetic Retinopathy
FBS: Fasting Blood Sugar
IDF: International Diabetic Federation
IQR: Inter Quartile Range
SBP: Systolic Blood Pressure
T2DM: Type Two Diabetes Mellitus
UGCSH: University of Gondar Comprehensive Specialized Hospital
WHO: World Health Organization
